# Screening for Highly Transduced Genes in Staphylococcus aureus Revealed Both Lateral and Specialized Transduction

**DOI:** 10.1128/spectrum.02423-21

**Published:** 2022-02-09

**Authors:** Janine Zara Bowring, Yue Su, Ahlam Alsaadi, Sine L. Svenningsen, Julian Parkhill, Hanne Ingmer

**Affiliations:** a Department of Veterinary and Animal Sciences, University of Copenhagen, Copenhagen, Denmark; b Department of Biology, University of Copenhagen, Copenhagen, Denmark; c Department of Veterinary Medicine, University of Cambridge, Cambridge, United Kingdom; Emory University School of Medicine

**Keywords:** *Staphylococcus aureus*, bacteriophages, gene transfer, mobile genetic elements, phages, transducing particles, transduction, transposons

## Abstract

Bacteriophage-mediated transduction of bacterial DNA is a major route of horizontal gene transfer in the human pathogen, Staphylococcus aureus. Transduction involves the packaging of bacterial DNA by viruses and enables the transmission of virulence and resistance genes between cells. To learn more about transduction in S. aureus, we searched a transposon mutant library for genes and mutations that enhanced transfer mediated by the temperate phage, ϕ11. Using a novel screening strategy, we performed multiple rounds of transduction of transposon mutant pools selecting for an antibiotic resistance marker within the transposon element. When determining the locations of transferred mutations, we found that the screen had selected for just 1 or 2 transposon mutant(s) within each pool of 96 mutants. Subsequent analysis showed that the position of the transposon, rather than the inactivation of bacterial genes, was responsible for the phenotype. Interestingly, from multiple rounds, we identified a pattern of transduction that encompassed mobile genetic elements as well as chromosomal regions both upstream and downstream of the phage integration site. The latter was confirmed by DNA sequencing of purified phage lysates. Importantly, transduction frequencies were lower for phage lysates obtained by phage infection rather than induction. Our results confirmed previous reports of lateral transduction of bacterial DNA downstream of the integrated phage but also indicated a novel form of specialized transduction of DNA upstream of the phage. These findings illustrated the complexity of transduction processes and increased our understanding of the mechanisms by which phages transfer bacterial DNA.

**IMPORTANCE** Horizontal transfer of DNA between bacterial cells contributes to the spread of virulence and antibiotic resistance genes in human pathogens. For Staphylococcus aureus, bacterial viruses play a major role in facilitating the horizontal transfer. These viruses, termed bacteriophages, can transfer bacterial DNA between cells by a process known as transduction, which despite its importance is only poorly characterized. Here, we employed a transposon mutant library to investigate transduction in S. aureus. We showed that the genomic location of bacterial DNA relative to where bacteriophages integrated into that bacterial genome affected how frequently that DNA was transduced. Based on serial transduction of transposon mutant pools and direct sequencing of bacterial DNA in bacteriophage particles, we demonstrated both lateral and specialized transduction. The use of mutant libraries to investigate the genomic patterns of bacterial DNA transferred between cells could help us understand how horizontal transfer influences virulence and resistance development.

## INTRODUCTION

S. aureus is a Gram-positive opportunistic pathogen that causes a wide range of diseases in humans. It can become resistant to a variety of antibiotics like methicillin, tetracycline, and vancomycin, and resistance is limiting treatment options (1, 2). The staphylococcal genome is highly plastic, largely due to horizontal gene transfer mediated by mobile genetic elements (MGEs), such as staphylococcal pathogenicity islands (SaPIs), plasmids, and phages (3–5). Phage-mediated transduction appears to be an important route of gene transfer (4, 6). Transduction is a process whereby some phages can package bacterial DNA at a low frequency and transfer this DNA between cells (6, 7). Transducing phages are often temperate because they can both be integrated into the bacterial genome as prophages (lysogeny) or be lytic. Transduction can occur through several different mechanisms namely, generalized, specialized, and lateral transduction. In S. aureus, generalized transduction has been used for many years as a genetic tool and recently, the mechanism for lateral transduction was established (8–10). In contrast, we know little about specialized transduction in this organism.

Phage ϕ11 is one of the best-characterized staphylococcal phages and is regularly used as a laboratory tool for transferring genes by transduction (11). Phage ϕ11 is a *pac*-type phage, meaning that it recognizes a phage ‘*pac*’ site and packages DNA into the capsid in a ‘headful’ manner, terminating when the capsid is filled with slightly more than the ∼45 kb phage genome (11, 12). As such, ϕ11 can transfer bacterial DNA by generalized transduction when bacterial DNA contains sequences with homology to the phage ‘*pac’* sites (11, 13, 14).

Lateral transduction is a recently identified form of phage-mediated transduction in both S. aureus and Salmonella (10, 15). Here, it was found that for some temperate phages, replication begins before excision from the host chromosome leading to the replication and packing of the integrated prophage together with the flanking regions of bacterial DNA. In practice, this has led to transduction of up to 100 kb of bacterial DNA downstream of the prophage with frequencies of 1000-fold higher compared to generalized transduction. The study identified that this effect was unidirectional from the *pac* site and so the region upstream of the phage was not transferred (10).

Specialized transduction occurs when a prophage excises incorrectly from the bacterial chromosome (16–25). Instead of a precise excision of the full phage genome between the att*_L_* and att*_R_* sites, an incomplete portion of the phage excises alongside some of the flanking bacterial DNA either upstream or downstream of the phage. If the phage *pac* site is present, this hybrid DNA will be replicated, packaged into phage capsids, and transduced (16, 18, 21). It is limited to the immediate regions of bacterial DNA flanking a prophage. Specialized transduction mechanisms have been reported for phages infecting several bacterial genera, including Bacillus, Salmonella, Pseudomonas, and Vibrio (16, 17, 20, 23). However, literature is absent on specialized transduction by staphylococcal phages.

In a recent publication, DNA short- and long-read sequencing was used to investigate the patterns of bacterial DNA in transducing particles in different genera and species of bacteria (26). The patterns observed indicated that different phages and prophages are either more or less prone to lateral, specialized, or generalized transduction and that the same temperate phage may transduce bacterial DNA differently depending on whether that phage is undergoing lytic infection or lysogenic induction (27).

To understand more about transduction processes in S. aureus, we explored a screening methodology that allowed us to identify S. aureus transposon mutants that were preferentially transduced from a pool of mutants. We utilized the Nebraska transposon mutant library (NTML) in S. aureus strain JE2 (28, 29), infecting pools of 96 mutants with the transducing, lysogenic phage ϕ11. From this screen, we anticipated identifying genes that either, when inactivated, promoted transduction or were located within the chromosome in regions transduced at higher rates than elsewhere. We showed that the transposon mutations in the chromosomal regions flanking the phage integration site were transduced at higher rates by both lateral and specialized transduction. This study established a simple screening method that reveals patterns of transduction at the genome level, which can be readily applied to transposon mutant libraries of other bacteria. Remarkably, this study was the first to demonstrate specialized transduction in S. aureus.

## RESULTS

### Transduction selection preferentially identifies transposon mutations in MGEs and regions flanking prophage ϕ11.

**FIG 1:**
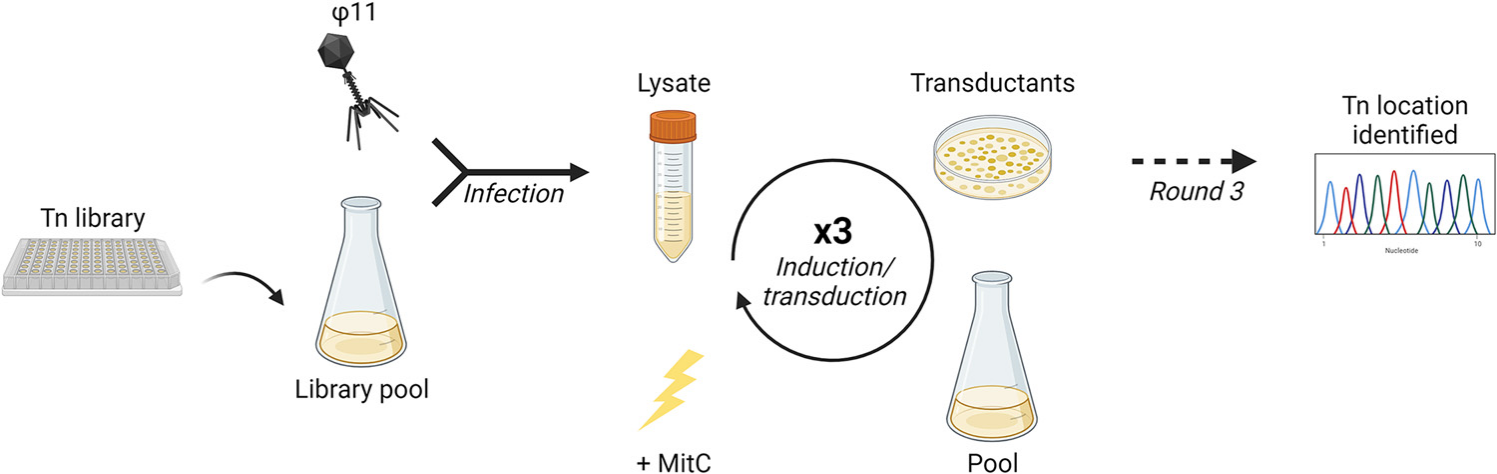
Schematic drawing of the experimental set-up. Depiction of the experimental set-up, indicating the initial pooling and infection by φ11 of the NARSA transposon mutant library plates, followed by successive rounds of transduction into 8325-4 φ11, pooling of the resulting transductants, and induction of the φ11 prophage to produce the next round’s lysate. At round three, the resulting transductants were processed as previously described to identify the location of transposon insertion (28, 34). Created with BioRender.com.

Intending to identify genes or mutations that increase transduction frequency, we screened the S. aureus JE2 NTML for mutations that were preferentially transduced. To this end we carried out consecutive rounds of transduction on pools of 96 library mutants, infecting these with the transducing temperate phage ϕ11 to produce an ‘infection’ phage lysate containing transducing particles (Fig. 1). We then performed transduction using these lysates with the laboratory strain 8325-4 ϕ11, a strain containing only the prophage φ11, as the recipient. Having the ϕ11 prophage present in the recipient, rather than using the phage-less 8325-4, prevented phage superinfection and, thus, increased survival of the transductants. Likewise, the use of this recipient prevented a mixed population of 8325-4 transductants and 8325-4 φ11 transductants, which would occur using an 8325-4 recipient due to the integration of φ11. The transfer from JE2 to the 8325-4 φ11 was conducted to exclude interference from the prophages USA300φSa2 and USA300φSa3, present in JE2. The core genomes of JE2 and 8325-4 have an identity of 96.6% when aligned using MAUVE, and there was no difference in φ11 phage titer between 8325-4 and JE2 recipients (Fig. S1).

Transposon-containing transductants in 8325-4 φ11 were selected for erythromycin resistance, encoded by the transposon. These transductants were pooled and the ϕ11 prophage was induced to produce a ‘round 1 induction’ lysate. The transduction and induction steps were repeated to produce ‘round 2’ and ‘round 3′ transduction lysates. Although this was necessary for the selection of highly transduced mutations, this could also have impacted our screen by selecting for transposon mutants of greater fitness. At each stage, the colony forming unit (CFU)/mL and plaque forming unit (PFU)/mL were recorded, and the transduction frequency was calculated, controlling for phage variation ([CFU/mL]/[PFU/mL]; Fig. S2). Importantly, the transduction frequencies showed a significant increase over the three rounds of transduction screening for the seven 96-well plates examined, with the overall mean increasing from −6.8 at infection to −5.7, −4.5, and −3.7 at rounds 1, 2, and 3, respectively (Fig. S2).

**FIG 2:**
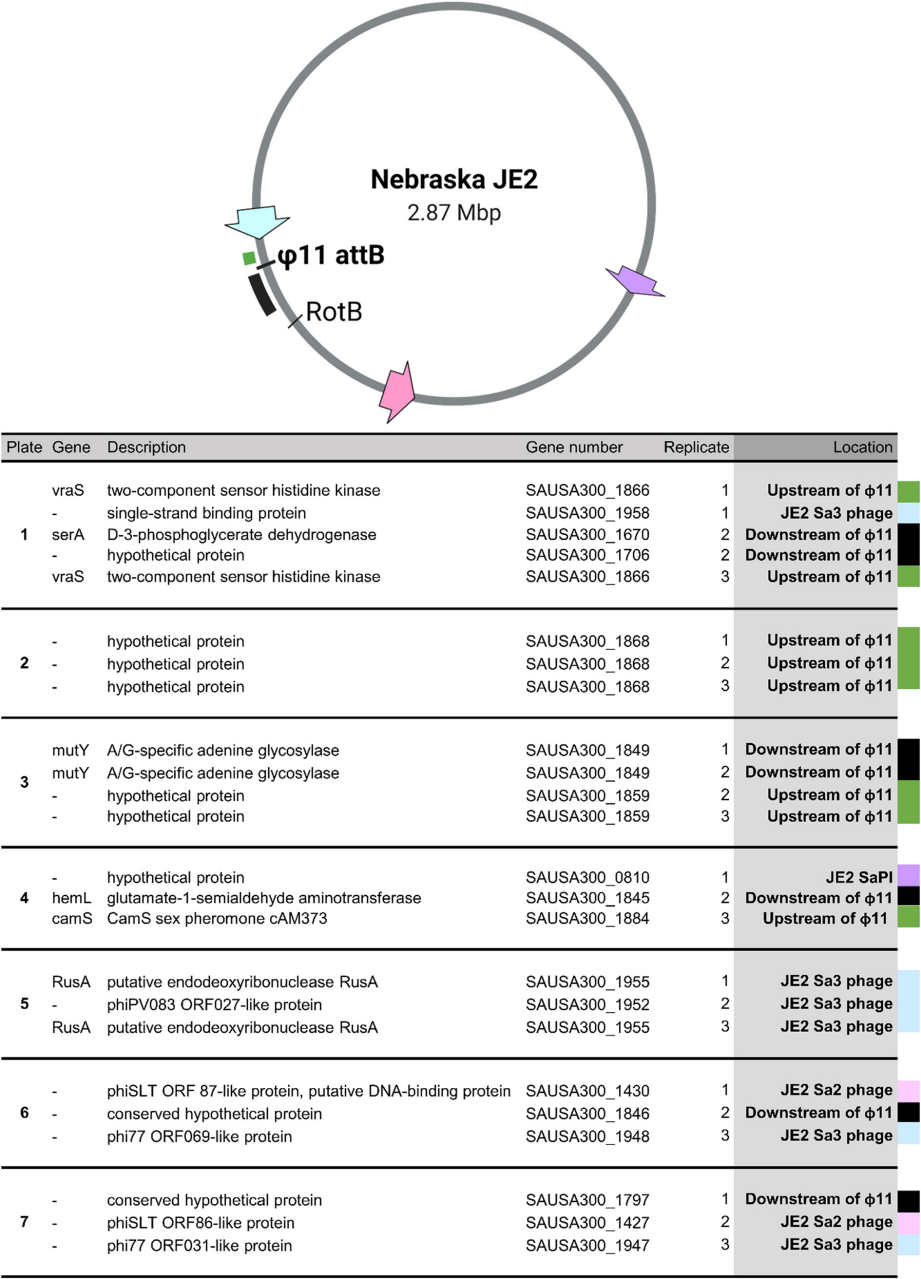
Locations of the identified transposon mutations in the JE2 genome. Depiction of NTML JE2 genome (NCBI accession number 007793.1), with MGEs USA300ϕSa2 (pink arrow), USA300ϕSa3 (blue arrow), and JE2 SaPI5 (purple arrow). The ϕ11 insertion site (att_B_) is indicated, as are the upstream and downstream regions flanking the att_B_ (green and black bars) and the position of the Tn_rotB control. The table shows the gene in which the transposon was inserted for each identified mutant. **‘**Plate’ refers to the plate of the pooled 96 mutants (see Table S1 for NARSA equivalent), ‘replicate’ refers to which biological replicate screen the mutation was identified in, ‘location’ identifies whether the transposon insertion was in an MGE-situated gene or the chromosome (Chr). Upstream and downstream refer to the position of the gene relative to the ϕ11 prophage insertion, using the prophage directionality (eg. downstream = downstream of the att_R_, upstream = upstream of the att_L_). Locations are color-coordinated with the genome map regions.

At the round 3 transduction, we collected 10 colonies from each plate pool and identified the locations of the transposon mutations as described (28). Most pools contained one predominant transposon mutant per biological replicate, leading to ≤4 being identified in total for each plate (Fig. 2). For the first biological replicate, only one transposon mutant was identified in 6 of the 7 pools while transposon mutants were identified for plates 1 and 2. Strikingly, half (4 out of 8) of the transposon mutations identified in our initial screen were situated on MGEs (Fig. 2). Of these, one transposon was in a SaPI5 gene, one in the USA300ϕSa2 prophage of JE2 while the other 2 were located in the USA300ϕSa3 JE2 prophage. These MGE-located transposon mutations were distributed throughout the chromosome (Fig. 2). The other 4 chromosomally situated non-MGE-related transposon mutations clustered around the integration site of the ϕ11 (att_B_; Fig. 2). As a prophage, φ11 integrates into an intergenic region of the 8325 chromosomes with a specific directionality, where the *attL* was situated at ∼1.967 Mb and the *attR* at ∼1.923 Mb (29). Relative to the ϕ11 prophage, 2 of the transposon mutations were situated upstream, but close to, the ϕ11 att_B_, while the other 2 were located downstream of this site. For subsequent biological replicates, the final transduction plate colonies were pooled for DNA extraction and transposon location identification, in most cases giving one predominant transposon mutant (Fig. 2). The transposon mutations identified were in some cases identical to the first replicate, while all others were in the same regions of either MGEs or upstream and downstream of the ϕ11 att_B_. To check the impact of fitness on our screen, we performed growth curves on two of the transposon mutants identified (Tn_1868 and Tn_1849) together with two mutants that were included in the pool but not found in the screen (Tn_1799 and Tn_1883) as well as 8325-4 and 8325-4 ϕ11 controls (Fig. S3). There were no significant differences in doubling the time between the screen-identified mutants and the nonidentified ones (Table S3). Therefore, we hypothesized that we were identifying transposon mutations that were preferentially transduced due to either induced MGE mobility or lateral and specialized transduction.

### Transposon mutants upstream and downstream of ϕ11 have an increased transduction frequency.

**FIG 3:**
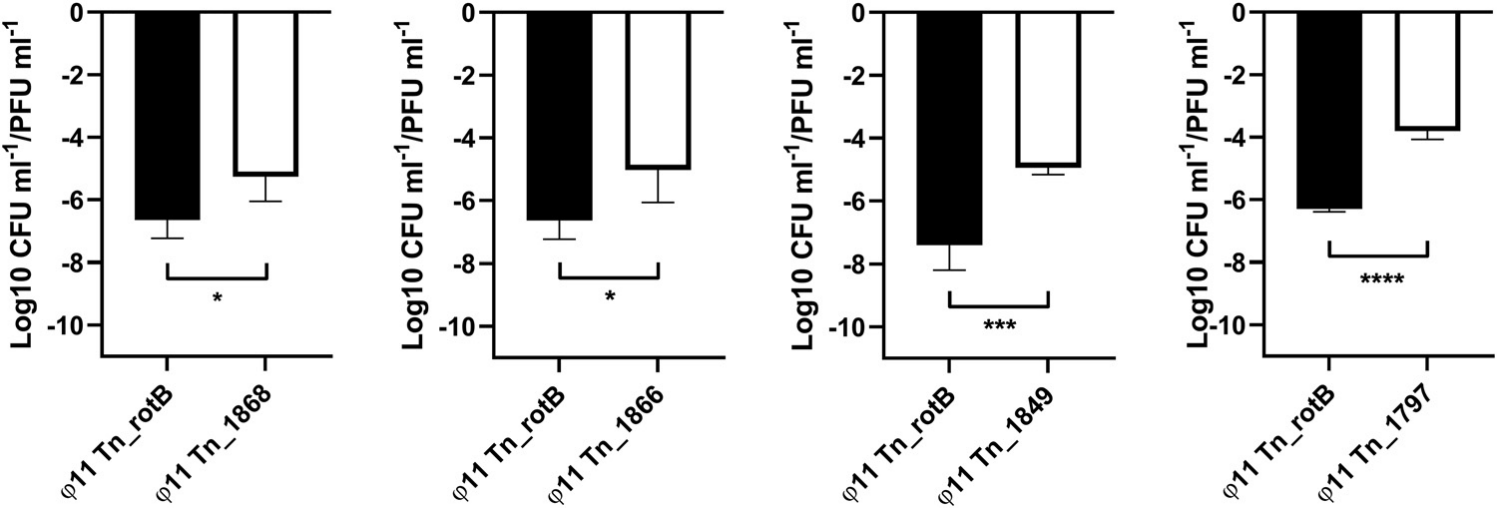
The transduction frequency of the transposon mutants located upstream and downstream of the ϕ11 integration site. Graphs showing the transduction frequency (log-transformed CFU mL^−1^/PFU mL^−1^) for each mutant compared with Tn_rotB (mean and SD, 4 biological replicates). Unpaired t tests were performed and *P* values from left to right were 0.0312*, 0.0346*, 0.0008***, <0.0001****.

**FIG 4:**
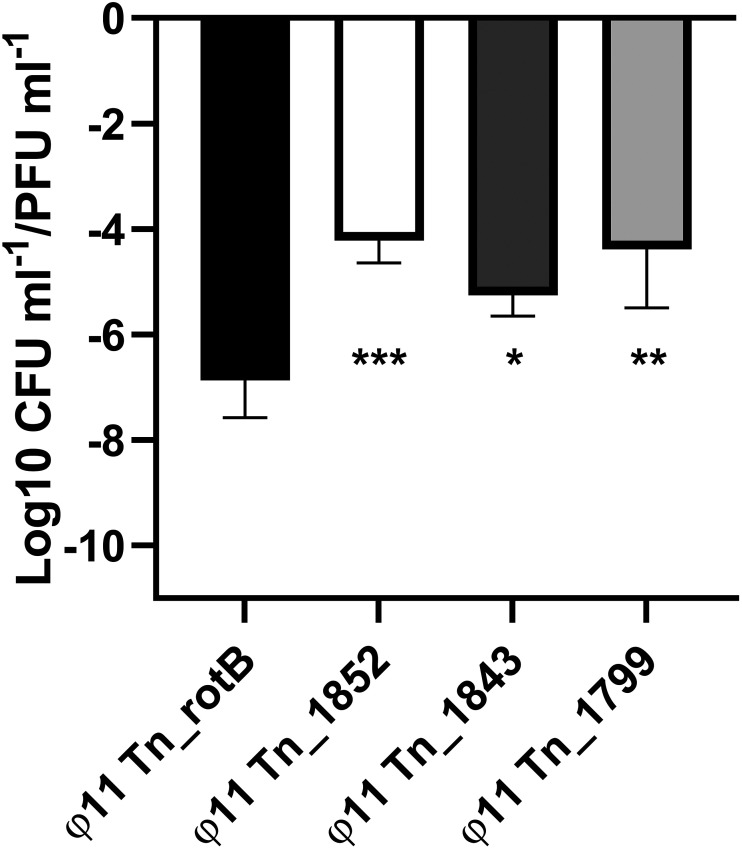
The transduction frequency of chromosomal regions downstream of the ϕ11 integration site was not observed in the original transduction screen. The graph shows the CFU/PFU transduction frequency (mean and SD, 4 replicates) for the induced lysate of each downstream mutant compared with Tn_rotB control. A one-way ANOVA was performed with Dunnett's multiple-comparison test comparing the mean of the Tn_rotB control with each mutant's mean, and *P* values from left to right were 0.0006***, 0.0203*, and 0.0010**.

**FIG 5:**
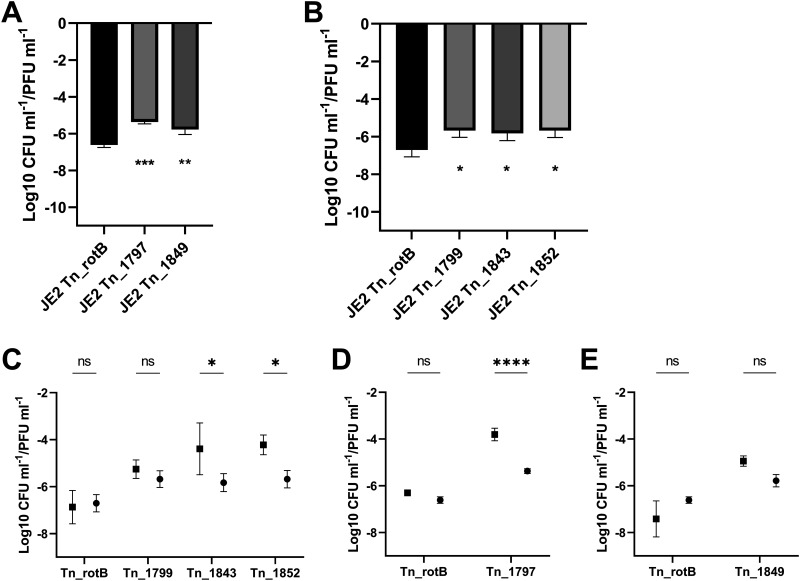
Lateral transduction of JE2 transposon mutations downstream of ϕ11. (A) The CFU/PFU transduction frequency following infection with ϕ11 for each JE2 downstream mutant from the initial screen compared with JE2 Tn_rotB (Tn inserted into SAUSA300_1797 and SAUSA300_1849, respectively). *P* values for a one-way analysis of variance (ANOVA) with Dunnett's multiple-comparison test were 0.0003*** and 0.0027** (left to right). (B) The CFU/PFU transduction frequency for the control JE2 Tn_rotB and the other JE2 Tn mutants downstream of the ϕ11 integration site following infection with ϕ11 (Tn inserted into SAUSA300_1799, SAUSA300_1843, and SAUSA300_1852, respectively.) *P* values for a one-way ANOVA with Dunnett's multiple-comparison test were 0.0233*, 0.0476*, and 0.0233* (left to right). All values shown werelog-transformed and showed the means and SD from the 3 replicate values. (C to E) The CFU/PFU transduction frequency following either infection or induction with ϕ11. (C) Comparison of the induction (square) and infection (circle) transduction frequencies of the neighboring transposon mutants and the Tn_rotB control, showing the means and standard deviation (SD). *P* values for a two-way ANOVA with Sidak multiple-comparison test were 0.9944^ns^, 0.8400 ^ns^, 0.0212*, and 0.0190* left to right. (D and E) Comparison of the induction (square) and infection (circle) transduction frequencies of the Tn_rotB control together with Tn_1797 and Tn_1849, respectively. *P* values for a two-way ANOVA with Sidak multiple-comparison test were (D) 0.0830 ^ns^ and <0.0001**** (E) 0.0874^ns^, and 0.0747^ns^.

**FIG 6:**
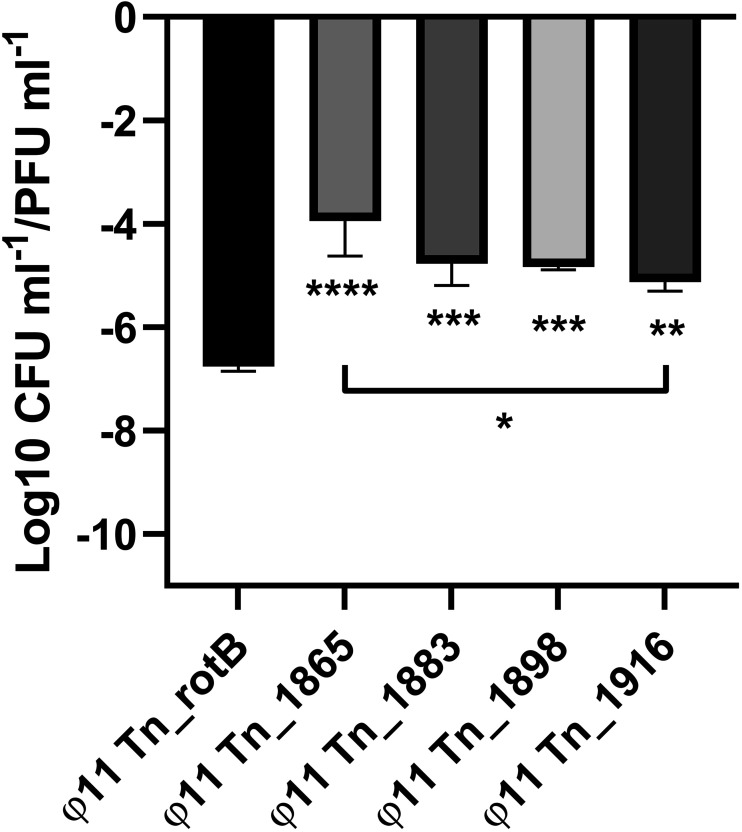
The transduction frequency of transposon mutations from the chromosomal region upstream of the ϕ11 integration site that was not selected in the original transduction screen. The graph shows the CFU/PFU transduction frequency for each upstream mutant compared with Tn_rotB. A one-way ANOVA was performed with Tukey multiple-comparison test comparing the means. Comparison of the Tn_rotB control with each mutant mean gave *P* values from left to right of <0.0001 ***, 0.0005 ***, 0.0006***, and 0.0022*. An additional comparison of the Tn mutant closest to the ϕ11 insertion site (Tn_1865) to the one furthest away (Tn_1916) is indicated by the black line, with a *P* value of 0.0188*. All values shown were log-transformed and showed the means and SD for 3 biological replicates.

**FIG 7:**
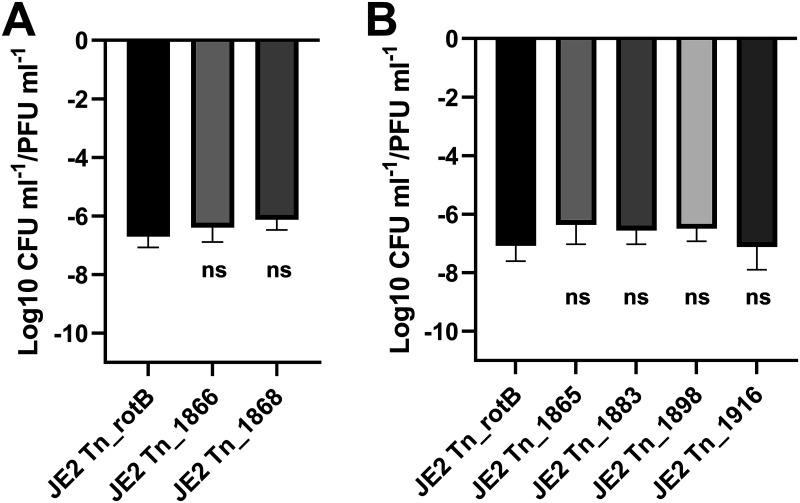
The transduction frequency of upstream-located JE2 transposon mutations following infection with ϕ11. (A) The CFU/PFU transduction frequency for the control JE2 Tn_rotB and the different screen-identified Tn mutants upstream of the ϕ11 integration site following infection with ϕ11 (Tn inserted into SAUSA300_1866 and SAUSA300_1868, respectively). *P* values for a one-way ANOVA with Dunnett's multiple-comparison test were 0.5807^ns^ and 0.2225^ns^. (B) The CFU/PFU transduction frequency for the control JE2 Tn_rotB and the different neighboring Tn mutations upstream of the ϕ11 integration site following infection with ϕ11 (Tn inserted into SAUSA300_1865, SAUSA300_1883, SAUSA300_1898, and SAUSA300_1916, respectively). *P* values for a one-way ANOVA with Dunnett's multiple-comparison test were 0.4197^ns^, 0.6682^ns^, 0.5882^ns^, and 0.9999^ns^ (left to right). All values shown were log-transformed and showed the means and standard deviation from the 3 replicate values.

The chromosomal transposon mutants clustering around the ϕ11 att_B_ interested us as potential incidents of specialized or lateral transduction (replicate 1: SAUSA300_1866, SAUSA300_1868, SAUSA300_1849, and SAUSA300_1797). Therefore, we transduced these individual transposon mutations into 8325-4 ϕ11 and confirmed by multiplex PCR targeting the phage integrases that none of the JE2 endogenous phages had been transferred (30). Using these ‘clean background’ strains, we confirmed that the transduction frequencies in the 4 chromosomal transposon mutants were higher than our 8325-4 ϕ11 *rotB*::erm transposon control. The *rotB* transposon mutant has a wild-type phenotype in respect to bacterial growth and transduction, and ϕ11 should only transfer this transposon mutation by generalized transduction based on its position in the 8325 genomes (1.794 Mb; Fig. 2). All transposon mutants had significantly higher transduction frequencies than the *rotB* control (Fig. 3). The initial data (CFU/mL and PFU/mL) for these experiments can be seen in Fig. S4 and a schematic showing the locations of the transposon mutants used for Fig. 3 to 7 can be observed in Fig. S5.

### Transposon mutations downstream of the ϕ11 integration site are transferred by lateral transduction.

Next, we defined the type of transduction occurring with these 4 chromosomal transposon mutants. Lateral transduction occurs in bacterial DNA up to 100 kb downstream of a laterally transducing prophage based on the pac site encoded by that phage. In the case of ϕ11, the *pac* site was in the small terminase gene and this meant that the region downstream of the att_R_ (<1.923 Mb) was transferred by lateral transfer while the region upstream of the att_L_ (>1.967 Mb) was not (11, 13). Therefore, we hypothesized that the downstream transposon mutations could be transduced laterally. To establish whether the higher transduction frequencies observed in the mutants with transposons located downstream of ϕ11 were due to the effects of the specific transposon insertions or the more general effect of lateral transduction, we investigated other transposon mutants from the same region of DNA. We used three different mutants where the transposon was located downstream of the prophage but had not been identified by the replicate 1 screen (Tn_1799, Tn_1843, and Tn_1852; Tn_1852 was subsequently identified in a later screen). We measured the transduction frequency of these transposon mutants in 8325-4 φ11 following induction. All three had a significantly higher transduction frequency than the Tn_rotB control (Fig. 4) and were comparable to the Tn_1849 and Tn_1797 mutants.

Because these mutants had a high transduction frequency similar to that of the mutants from the screen, it suggested that lateral transduction was responsible. Because lateral transduction only occurred when a prophage was induced, we tested the transduction frequency of both the original mutants and these ‘neighboring’ mutants following infection where lateral transduction could not occur. The transposon mutants in the JE2 background were infected with ϕ11 and the transduction frequency of the resulting lysate was established. The transduction frequencies of all the transposon elements located downstream of ϕ11 were higher than the Tn_rotB control (Fig. 5A and B). However, these transduction frequencies were significantly reduced for Tn_1843, Tn_1852, and Tn_1797 compared to the previous induction-linked frequencies (Fig. 5C to E). Furthermore, Tn_1799 and Tn_1849 both showed lower transduction frequencies following infection, although these differences were not significant. This showed that lateral transduction was the main cause of the increased transduction frequencies downstream of the ϕ11 integration site.

### Transposon mutations upstream of the ϕ11 integration site are transferred by specialized transduction.

To establish whether the genes located upstream and close to the ϕ11 integration site were transduced at higher frequencies due to gene-specific effects or because of specialized transduction, additional transposon mutants from this region were examined. The mutants in the same region as Tn_1866 and Tn_1868 showed comparable transduction frequencies, while the mutant with the transposon element furthest from the phage integration site (62 kb upstream) showed a gradual reduction in transduction frequency, albeit notably higher than the Tn_rotB control (Fig. 6; Tn_1865, Tn_1883, and Tn_1898 versus Tn_1916, respectively).

**FIG 8:**
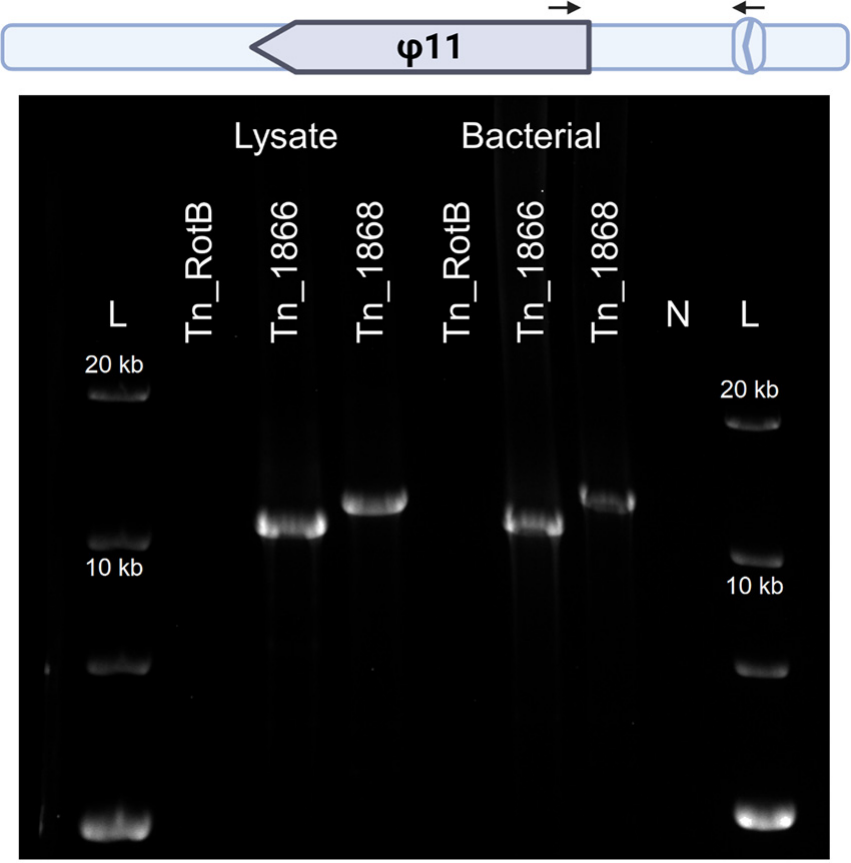
Amplified specialized transduction fragments from precipitated phage DNA. A 0.6% agarose gel showing the PCR products of a long-range amplification using TaKaRa LA polymerase. Diagram indicates the position of the ϕ11 phage genome in relation to the transposon mutations and black arrows indicate the primer positions. The reverse primer anneals to the erythromycin resistance gene present in all the transposon mutations. Templates, from left to right, were DNA extracted from precipitated phage capsids from an induced lysate of 8325-4 ϕ11 Tn_RotB, an induced lysate of 8325-4 ϕ11 Tn_1866, an induced lysate of 8325-4 ϕ11 Tn_1868 or bacterial DNA from a culture of 8325-4 ϕ11 Tn_rotB, 8325-4 ϕ11 Tn_1866, and 8325-4 ϕ11 Tn_1868. N indicates no template. The expected size for region ϕ11 to Tn_1866 was ∼10.6 kb and ∼11.7 kb for region ϕ11 to Tn_1868. Three biological replicates were performed, including induction, precipitation, DNA extraction, and PCR, and the above gel is representative. Primers Martn-ermR and Phage direction-3f were used for all templates (Table S2).

Furthermore, when antibiotic resistances from the original JE2 transposon mutants were transduced following ϕ11 infection, the transduction frequencies were lower and not significantly different to the Tn_rotB control (Fig. 7). Given that specialized transduction did not occur during infection, these results suggested that specialized transduction was occurring in the region immediately upstream of the ϕ11 and caused the higher levels of transduction seen. Indeed, by using primers annealing within the NTML transposon (Martn-ermR) and the ϕ11 phage (phi11-direction-3f) it was possible to amplify products from induced lysates of 8325-4 ϕ11 Tn_1866 and 8325-4 ϕ11 Tn_1868 (Fig. 8). This indicated that some transducing particles in these lysates contained the upstream regions of bacterial DNA alongside the 5′-end region of the phage genome, which would be expected for specialized transduction.

Likewise, when we transduced the Tn_1866 and Tn_1868 transposon mutations into an 8325-4 as opposed to 8325-4 ϕ11 recipient, the percentage of 8325-4 recipients that by PCR were also positive for the Sa5 Int encoded by ϕ11 was significantly higher than observed when examining cells where the Tn_RotB had been transduced (Fig. S6). This would be expected with specialized transduction where the majority of transductant DNA injected into a new recipient cell would recombine with an integrated coinfecting phage and, therefore, selecting for a higher proportion of lysogenic recipient cells (18).

### Regions identified from the preferentially transduced transposon elements are packaged at higher frequencies than the rest of the genome.

**FIG 9:**
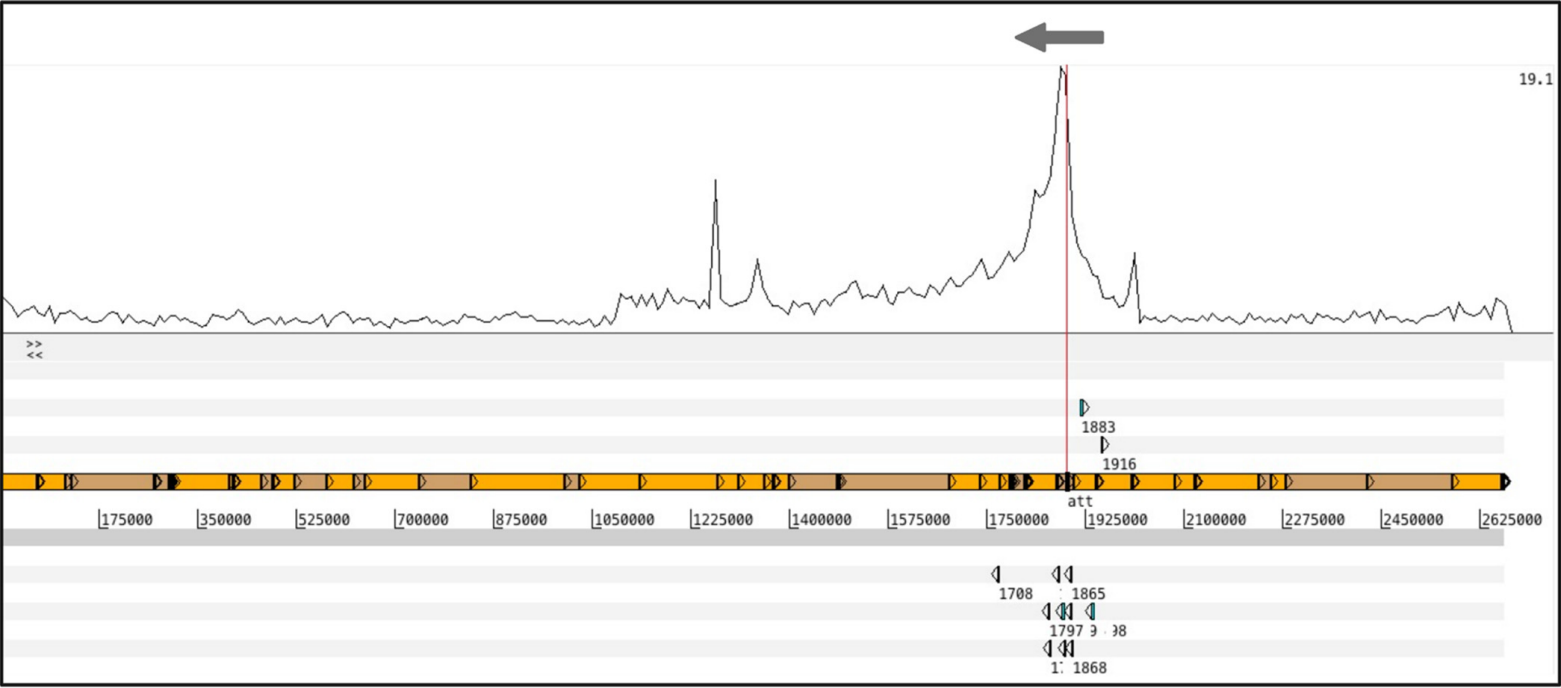
Mapping of lysate-derived DNA to the 8325-4 chromosome. The top graph shows read mapping to the 8325-4 chromosome. Elevated coverage was identified between 1093501 and 2011500 bp with an independent *t* test performed to compare the mean read mapping between the high coverage and the rest of the genome, *P* value <0.0001****. One-sample t tests were also performed to compare the mean read mapping at the boundaries of the region of elevated coverage. Bin 82 (1093501 to 1107000 bp) was compared against the mean of bins 1 to 81 (1 to 1093500 bp), *P* value <0.0001****, and bin 149 (1998001 to 2011500 bp) against the mean of bins 150 to 198 (2011501 to 2668682 bp), *P* value <0.0001****. The red line indicates the site of insertion of ϕ11 in the lysogen, with the gray arrow indicating the orientation of the phage. Beneath this is the six-frame translation (top three and bottom three lines) where the genes referred to in this study are represented by blue boxes, with the NTML gene numbers included. The central two lines represent the forward and reverse DNA strands, with the concatenated assembly contigs identified with alternating orange and brown boxes. The narrow peak of read mapping to the left of the insertion site is caused by mismapping of phage reads, and the narrow peak to the right is caused by multiple mapping to an rRNA operon.

To support our genetic screen, we sequenced phage ϕ11 lysates from mitomycin C induced 8325-4 ϕ11 lysogen to directly identify bacterial DNA packaged in phage capsids. Fig. 9 shows the mapping of the reads to 8325-4, thus removing reads mapping to the phage and allowing the lower frequency reads that mapped to the bacterial genome to be seen. Regions of elevated coverage were identified by dividing the genome into 13.5 Kbp bins and comparing the average coverage for each bin. The region between 1.09 and 2.01 Mbp was identified as the single region with significantly elevated coverage, with a mean depth overall of 5.6. This corresponded to the regions upstream and downstream of the ϕ11 att_B_ (indicated by the red line), supporting our screening data in which the identified mutants were clustered in this same region. The rest of the genome showed only the baseline level of reads mapping, with an average mean depth overall of 1.1. The pattern of the downstream bacterial DNA indicates that lateral transduction may be responsible, with a gradual reduction of mapped reads from high numbers near to the att_B_, decreasing further away from the attachment site. Lateral transduction has been shown to taper off in this fashion, while still facilitating the transfer of ∼200 kb of downstream bacterial DNA (10). Here, we see elevated read mapping as far away as 798 kb downstream from the att_B_ site (mean depth 1093501 to 1107000 bp, 2.6). The read alignments of the upstream bacterial DNA peak encompass 117 kb of DNA upstream of the att_B_ (mean depth 1998001 to 2011500 bp, 5.6). These reads could represent the effect of specialized transduction following the incorrect excision of the phage, albeit representing a larger region than one would expect with a phage genome length of ∼45 kb. In addition, there was mismapping of phage reads forming a large peak left of the att_B_ and a single large peak to the right of the att_B_ mapped to the ribosomal operon containing three ribosomal genes encoding 5S, 23S, and 16S rRNA, these were excluded from the analysis.

Based on our observations that these upstream regions were being transduced alongside the phage genome (Fig. 8), together with the unexpectedly large regions of elevated read mapping of these upstream regions (Fig. 9), we hypothesized that the transduction of this region was more complex than the wrongful excision of the prophage. Traditionally specialized transduction is defined as misexcision where nonhomologous recombination of the incomplete phage and bacterial flank leads to their circularization and *pac*-based packaging. This would limit specialized transduction to the flanking regions less than a phage genome-length (∼45 kb, plus redundancy) away from either the att_L_ or att_R_. However, here we identified elevated reads mapping and increased transduction frequencies up to 117 kb upstream of the att_B_ (Fig. 6 and 9, respectively). Interestingly, outside the region of elevated reads, the transduction frequencies were not significantly different from the Tn_RotB control (Fig. S7).

We hypothesized that the extended regions of elevated transduction frequencies could be explained by homologous regions flanking the integrated prophage and upstream region enabling homology-based circularization of this region, including the phage. BLASTn of the nucleotide sequence up to 45 kb downstream of the att_B_ with the upstream region of elevated reads mapping, indicated a 3.1 kb region of 99% identity between a region at the end of the elevated reads mapping and another ∼18 kb downstream of the att_B_. If these regions were circularized it would include the complete prophage genome, which could facilitate packaging of both phage and bacterial DNA from the *pac* gene while integrated into this episome-like circular structure, similarly to the packaging of integrated DNA during lateral transduction. To confirm whether this circularization could occur, primers were designed to amplify the hypothetically circularized region. Following large plasmid extraction, we could amplify the product from both noninduced and induced 8325-4 ϕ11 (Fig. S8).

## DISCUSSION

We described a high-throughput method by which the mobile regions of bacterial genomes can be identified. By screening pooled transposon mutants, we were able to identify different modes of phage-related horizontal gene transfer, including lateral and specialized transduction. The method could be applied to any strain for which there is a transposon mutant library available. Before our screen, we anticipated obtaining two types of mutants; namely, those where the transposon was placed in a genome location transferred with higher frequency, for example, by lateral transduction and mutants that due to inactivation of a bacterial gene would lead to increased transduction efficacy. The results showed, however, that we primarily obtained mutants belonging to the first category and, in addition, transposon elements that had been inserted into MGEs. Thus, for three of the resulting transposon mutants from the first replicate screen, the transposon element was inserted in phages being endogenous to the JE2 strain, namely, the USA300ϕSa2 and USA300ϕSa3, and these had been transferred to the 8325-4 background. This phage transfer was not unexpected because prophages can be induced by an infecting phage and the 8325-4 strain encodes attachment sites for these phages (11, 12, 31).

For two of the transposon mutants preferentially selected in our first screen, the transposon elements were located downstream of the ϕ11 attachment site (SAUSA300_1849 and SAUSA300_1797). These mutants displayed high transduction frequencies following induction, compared to a control where the antibiotic resistance marker was located elsewhere in the bacterial genome, and this effect was reduced when the transfer was monitored following phage infection. We concluded that the increased transduction frequency for these mutations was dependent on the induction of an integrated ϕ11 and that they were transferred by lateral transduction (10). This notion was supported by bacterial DNA sequences obtained from induced phage lysates that indicated extensive packaging of bacterial DNA downstream of the integrated phage. Interestingly, these data implicate lateral transduction at an even greater distance downstream of the att_B_ site than has previously been suggested (10), with slightly elevated reads mapping up to 798 kb downstream.

It has been shown that temperate phages are capable of both generalized and specialized transduction (16, 32), but specialized transduction had not been reported in S. aureus until now. However, for the transposon mutants Tn_1866 and Tn_1868 identified in our screen, the transposon element was located immediately upstream of the ϕ11 integration site at a distance of 7.6 and 8.7 kb, respectively, thus placing it comfortably within a region where specialized transduction could be expected to occur. The increased transduction frequencies of Tn_1866 and Tn_1868 were eliminated when lysates were created by infection rather than induction, removing the possibility of generalized transduction. Equally, they would not be transferred by lateral transduction, which occurs downstream of the phage *pac* site (10). This left specialized transduction as the remaining, feasible explanation. Indeed, we were able to identify PCR products containing both ϕ11 and the transposon using precipitated phage lysate from the strains with Tn_1866 and Tn_1868. This indicated that some phage capsids contained both the att_L_ region of the phage together with the upstream flanking region up to 8.7 kb away. Further, the majority (91% and 86%, respectively) of 8325-4 Tn_1866 and 8325-4 Tn_1868 transductant colonies were positive for the φ11 integrase. For Salmonella phage P22 it has been seen that specialized transduction into a recipient bacterium is mediated by recombination with an integrated prophage (15, 18). Therefore, we expect that in our case, specialized transduction may involve recombination between a transducing particle carrying DNA upstream of the phage and a coinfecting phage during infection. These data, support that the upstream genes are transferring through specialized transduction.

The DNA sequencing of the precipitated phage particles from induced 8325-4 ϕ11 correlated well with the transduction frequencies of our selected transposon mutation strains. As such, we were initially confused by the extended region of elevated reads mapping to 117 kb upstream of the phage attachment site. While the transposon mutations in genes 1866 and 1868 sit well within the region of classical specialized transduction (less than 10 kb upstream of the att_B_), the mutation in Tn_1916 is 62 kb upstream and still showed significantly higher transduction frequencies than the Tn_RotB control. Although this correlated with DNA sequencing, it could not be transferred by classical specialized transduction. Outside of the region indicated by our sequencing data, we no longer saw the higher transduction frequencies. Using the DNA sequencing data, we identified the end of the elevated reads mapping and found that this region had a 3.1 kb homologous region with 99% identity downstream of the phage. We hypothesized and confirmed that this region could recombine and in doing so form a large circle of DNA that would include the complete ϕ11 prophage. We speculate that the homologous regions up and downstream of the ϕ11 could facilitate a new variation of specialized transduction involving circularization of both prophage and flanking regions. Future work into this interesting phenomenon will look into the molecular mechanism by which such circularization could take place and how it is related to phage replication and packaging.

A limitation of our screen is the coselection for transposon mutants with greater fitness. This may explain why within our pooled mutants we identified a limited number of transposon mutations from the highly transduced regions, even though others were present in the pool. The transposon mutations identified in the screen may well represent those of higher fitness relative to the other highly transduced transposon mutants from that pool.

This study acts as a proof of concept for using transposon mutant libraries to investigate which regions of the bacterial genome are highly mobile. We showed phage transfer, generalized, specialized, and lateral transduction between staphylococcal strains. While the setup employed here was used to identify highly transduced genes and chromosomal locations, it may also reflect what happens over time in the real world. The release of ϕ11 from lysogens promotes the acquisition of bacterial DNA by auto-transduction and resulting transductants will, as lysogens, be able to repeat the process (33). Horizontal gene transfer underpins the evolution and adaptability of S. aureus and approaches such as the one adopted here will help us increase our understanding of which regions are transferred and how.

## MATERIALS AND METHODS

### Bacterial strains and growth conditions.

Bacterial strains used in this study are listed in Table S1. S. aureus strains were grown in tryptic soy broth (TSB) and on tryptic soy agar (TSA) plates (Oxoid) with or without antibiotics as appropriate.

### Growth curves.

Bacterial growth curves were performed in TSB using the bioscreen C automated microbiology growth curve analysis system. Briefly, overnight cultures were subcultured in TSB to an optical density at 600 nm of 0.01 and 250 μL plated in each well of the bioscreen C honeycomb 100-well plates. The OD_600_ was recorded every 30 min for 24 h at 37°C with shaking, which was paused 5 s before each read. Growth curves were analyzed using the GrowthCurver R script and the t_gen (or doubling time) was compared between strains.

### Bacteriophage titer.

Phage titers were performed as previously described (10, 33). Briefly, recipient strains were grown to 0.35 OD_600_ and 100 μL aliquots of recipient mixed with 100 μL phage lysate, at different dilutions in phage buffer (10^0^ to 10^−8^; MgSO_4_ 1 mM, CaCl_2_ 4 mM, Tris-HCl pH 8 50 mM, NaCl_2_ 0.1 M). After 10 min incubation at room temperature, 3 mL of liquid PTA (phage top agar; Oxoid nutrient broth no. 2, agar 3.5% wt/vol) was added and the mixture was poured out on PB plates (phage base; nutrient broth no. 2, agar 7% wt/vol). Plates were incubated at 37°C overnight and plaques were counted.

### Bacteriophage transduction.

Phage transductions were performed as previously described (10, 33). Briefly, recipient strains were grown to 1.4 OD_600_, 4.4 mM CaCl_2_ added, and 1 mL aliquots of recipient mixed with 100 μL phage lysate, at different dilutions in phage buffer (10^0^ to 10^−4^). After 20 min incubation at 37°C, 3 mL of liquid TTA (transduction top agar; TSA, 50% agar) was added and the mix was poured out on selective TSA plates. Plates were incubated at 37°C for 24 h and colonies were counted.

The transduction frequency was calculated using the CFU/mL divided by the PFU/mL to control for variation between lysates.

### Bacteriophage infection and induction.

Bacteriophage infection was performed as previously described (33). Briefly, recipient bacteria were grown to 0.15 OD_600_ and centrifuged, before resuspending the pellet in 1:1 TSB and phage buffer. The culture was then infected with phage at a multiplicity of infection (MOI) of 1 and incubated at 30°C, 80 rpm until complete visual lysis, normally after 3 to 4 h. Lysates were then filtered using 0.22 μM filters and stored at 4°C.

Bacteriophage induction was performed as previously described (10, 33). Briefly, lysogens were grown to an OD_600_ of 0.1 to 0.2 and 2 μg/mL mitomycin C (Sigma, from Streptomyces caespitosus) was added to induce resident prophages. Cultures were then incubated at 30°C, 80 rpm until complete visual lysis. Lysates were filtered using 0.22 μM filters and stored at 4°C.

### NTML transposon transduction screening.

One 96-well plate was replicated and grown overnight in selective TSB (erythromycin, 5 μg/mL) before pooling 10 μL of each mutant in 100 mL selective TSB in a 500 mL flask. The culture was infected with ϕ11 (MOI 1) as described. The lysate was then used to transduce the NTML transposons to 8325-4 ϕ1, as described. The transduction plate of undiluted lysate was harvested, pooled, and grown in selective TSB and the culture was diluted then induced. This lysate was used to transduce into the 8325-4 ϕ11 recipient a second time, and the transductants were pooled and induced. From the resulting lysate, a third transduction was performed, 10 transductant colonies selected, or all colonies were pooled, and the location of the transposon mutation(s) identified as previously described (28, 34).

### DNA sequencing.

Preparation of the phage lysates for DNA sequencing was done by precipitating the phage capsids before DNA extraction. For the phage precipitation, DNase (2.5 U/mL, Thermo) and RNase (1 μg/mL, Sigma) were added to the filtered lysate before incubation at 37°C for 1 h. NaCl_2_ (1 M) was added and mixed on the ice at 4°C for 1 h. The lysate was centrifuged at 11,000 × *g* for 10 min at 4°C and the supernatant was collected, mixed with PEG 8000 (10% wt/vol), before incubation on ice overnight at 4°C. The lysate was centrifuged at 11,000 × *g* for 10 min at 4°C and supernatant discarded, with falcon tubes dried. The pellet appeared as two vertical white lines and was resuspended in 8 mL phage buffer per 500 mL lysate, with resuspension for 1 h at 4°C. DNA extraction was performed using the MagAttract HMW DNA kit (Qiagen) per the manufacturer’s instructions. DNA concentration and absorbance peaks were checked using a Thermo NanoDrop 2000 and a Qubit Flex Fluorometer. A draft genome for 8325-4 was generated by sequencing chromosomal DNA on an Illumina miSeq following the manufacturer’s instructions. The sequence was assembled, and the contigs were ordered using the Sanger Institute pipelines (35). Phage lysate DNA was sequenced in the same way, and the resulting reads were mapped to this genome using SMALT (35, 36) and visualized using Artemis (35, 37).

To identify regions of elevated coverage, the genome was split into 198 bins of 13.5 Kbp, and an area of elevated coverage was identified between 1093501 and 2011500 bp, corresponding to bins 82 to 149, after calculating the coverage for each bin. To test whether this region had significantly elevated coverage compared to the rest of the genome, an independent *t* test was performed comparing the coverage observed in these two bin categories. Further statistical analyses were performed to test whether the elevated coverage at the ends of the elevated region remained above the background coverage of the rest of the genome. A one-sample *t* test was performed to compare the mean of the coverage distribution of bins 1 to 81 against bin 82, and another to compare bin 149 with the mean of bins 150 to 198.

### DNA methods.

General DNA manipulations were performed using standard procedures. Primers used in this study can be found in Table S2. PCRs were performed using DreamTaq Green master mix (2×; Thermo Fisher Scientific) per the manufacturer’s instructions. PCRs for identification of transposon locations were performed as previously described (28). PCR products were sequenced at Eurofins MWG Operon.

For identification of prophages in strains, a multiplex PCR for the main integrases of the staphylococcal phages was performed, as previously described (30). Initial screening of colonies was performed using 1 μL of template from a single colony resuspended in 50 μL ddH_2_O, boiled for 10 min, and on ice for 10 min. Results were confirmed using DNA template (10 ng), extracted using GenElute™ Bacterial Genomic DNA kit as per manufacturer’s instructions (Sigma-Aldrich). For integrase checks of transductants, colony PCRs using just the Sa5 Int primers was performed as described above.

For long amplifications, the Takara LA polymerase PCR kit was used per the manufacturer’s instructions with the following alteration, a 30 s annealing step was added at 55°C. Templates used were either bacterial DNA extracted with the GenElute™ Bacterial Genomic DNA kit or phage DNA extracted from phage lysates that had been precipitated, as previously described, and extracted using the GenElute™ Bacterial Genomic DNA kit with an extended proteinase K incubation of 1 h (Sigma-Aldrich). Fifty nanograms of the template were used for each PCR. The resulting products were run out on a 0.7% agarose gel at 30 V overnight.

For extraction of the large plasmid-like structures, samples were grown for 1 h, before induction with MitC as described. Samples were taken at the time of induction (T0) and 1 h after induction, with noninduced samples also taken. Fifty milliliters of the culture was taken per sample and prepared as previously described (38), with the amounts doubled and RNase added to the resuspension buffer. Sixty nanograms of the template were used for subsequent PCRs using a DreamsTaq polymerase kit.

### Statistical analyses.

Data analyses were performed in GraphPad Prism 8 software. Specific statistical tests are indicated in the figure legends, where appropriate.

## References

[1] Appelbaum PC. 2006. The emergence of vancomycin-intermediate and vancomycin-resistant Staphylococcus aureus. Clin Microbiol Infect 12:16–23. doi:10.1111/j.1469-0691.2006.01344.x. 16445720

[2] DeLeo FR, Otto M, Kreiswirth BN, Chambers HF. 2010. Community-associated methicillin-resistant Staphylococcus aureus. Lancet 375:1557–1568. doi:10.1016/S0140-6736(09)61999-1. 20206987 PMC3511788

[3] Goerke C, Wolz C. 2004. Regulatory and genomic plasticity of Staphylococcus aureus during persistent colonization and infection. Int J Med Microbiol 294:195–202. doi:10.1016/j.ijmm.2004.06.013. 15493830

[4] Lindsay JA. 2014. Staphylococcus aureus genomics and the impact of horizontal gene transfer. Int J Med Microbiol 304:103–109. doi:10.1016/j.ijmm.2013.11.010. 24439196

[5] Martínez-Rubio R, Quiles-Puchalt N, Martí M, Humphrey S, Ram G, Smyth D, Chen J, Novick RP, Penadés JR. 2017. Phage-inducible islands in the Gram-positive cocci. ISME J 11:1029–1042. doi:10.1038/ismej.2016.163. 27959343 PMC5363835

[6] Xia G, Wolz C. 2014. Phages of Staphylococcus aureus and their impact on host evolution. Infect Genet Evol 21:593–601. doi:10.1016/j.meegid.2013.04.022. 23660485

[7] Deghorain M, Van Melderen L. 2012. The Staphylococci Phages Family: an Overview. Viruses 4:3316–3335. doi:10.3390/v4123316. 23342361 PMC3528268

[8] Lennox ES. 1955. Transduction of linked genetic characters of the host by bacteriophage P1. Virology 1:190–206. doi:10.1016/0042-6822(55)90016-7. 13267987

[9] Zinder ND, Lederberg J. 1952. Genetic exchange in Salmonella. J Bacteriol 64:679–699. doi:10.1128/jb.64.5.679-699.1952. 12999698 PMC169409

[10] Chen J, Quiles-Puchalt N, Chiang YN, Bacigalupe R, Fillol-Salom A, Chee MSJ, Fitzgerald JR, Penadés JR. 2018. Genome hypermobility by lateral transduction. Science 362:207–212. doi:10.1126/science.aat5867. 30309949

[11] Iandolo JJ, Worrell V, Groicher KH, Qian Y, Tian R, Kenton S, Dorman A, Ji H, Lin S, Loh P, Qi S, Zhu H, Roe BA. 2002. Comparative analysis of the genomes of the temperate bacteriophages φ11, φ12 and φ13 of Staphylococcus aureus 8325. Gene 289:109–118. doi:10.1016/S0378-1119(02)00481-X. 12036589

[12] Gillaspy AF, Worrell V, Orvis J, Roe BA, Dyer DW, Iandolo JJ, 2014. The Staphylococcus aureus NCTC 8325 Genome, p 381–412. In Fischetti VA, Novick RP, Ferretti JJ, Portnoy DA, Rood JI. (ed), Gram-Positive Pathogens, 2nd ed, vol3. ASM Press, Washington, DC.

[13] Lander GC, Tang L, Casjens S, Gilcrease EB, Prevelige PE, Poliakov A, Potter CS, Carragher B, Johnson JE. 2006. The structure of an infectious P22 virion shows the signal for headful DNA packaging. Science 312:1791–1795. doi:10.1126/science.1127981. 16709746

[14] Schmieger H. 1982. Packaging signals for phage P22 on the chromosome of Salmonella typhimurium. Mol Gen Genet 187:516–518. doi:10.1007/BF00332637. 6757664

[15] Fillol-Salom A, Bacigalupe R, Humphrey S, Chiang YN, Chen J, Penadés JR. 2021. Lateral transduction is inherent to the life cycle of the archetypical Salmonella phage P22. Nat Commun 12:6510. doi:10.1038/s41467-021-26520-4. 34751192 PMC8575938

[16] Kwoh DY, Kemper J. 1978. Bacteriophage P22-mediated specialized transduction in Salmonella typhimurium: high frequency of aberrant prophage excision. J Virol 27:519–534. doi:10.1128/JVI.27.3.519-534.1978. 359827 PMC525839

[17] Zahler SA, Korman RZ, Rosenthal R, Hemphill H. 1977. Bacillus subtilis bacteriophage SPβ: localization of the prophage attachment site, and specialized transduction. J Bacteriol 129:556–558. doi:10.1128/jb.129.1.556-558.1977. 401505 PMC234961

[18] Kwoh DY, Kemper J. 1978. Bacteriophage P22-mediated specialized transduction in Salmonella typhimurium: identification of different types of specialized transducing particles. J Virol 27:535–550. doi:10.1128/JVI.27.3.535-550.1978. 359828 PMC525840

[19] Mackey CJ, Zahler SA. 1982. Insertion of bacteriophage SP beta into the citF gene of Bacillus subtilis and specialized transduction of the ilvBC-leu genes. J Bacteriol 151:1222–1229. doi:10.1128/jb.151.3.1222-1229.1982. 6809729 PMC220399

[20] Cavenagh MM, Miller RV. 1986. Specialized transduction of Pseudomonas aeruginosa PAO by bacteriophage D3. J Bacteriol 165:448–452. doi:10.1128/jb.165.2.448-452.1986. 3080405 PMC214439

[21] Hoppe I, Roth J. 1974. Specialized transducing phages derived from salmonella phage P22. Genetics 76:633–654. doi:10.1093/genetics/76.4.633. 4599252 PMC1213093

[22] Porter RD, Lark MW, Low KB. 1981. Specialized transduction with lambda plac5: dependence on recA and on configuration of lac and att lambda. J Virol 38:497–503. doi:10.1128/JVI.38.2.497-503.1981. 6454007 PMC171180

[23] Campos J, Martínez E, Marrero K, Silva Y, Rodríguez BL, Suzarte E, Ledón T, Fando R. 2003. Novel Type of Specialized Transduction for CTXϕ or Its Satellite Phage RS1 Mediated by Filamentous Phage VGJϕ in Vibrio cholerae. J Bacteriol 185:7231–7240. doi:10.1128/JB.185.24.7231-7240.2003. 14645284 PMC296256

[24] Fink PS, Zahler SA. 1982. Specialized transduction of the ilvD-thyB-ilvA region mediated by Bacillus subtilis bacteriophage SP beta. J Bacteriol 150:1274–1279. doi:10.1128/jb.150.3.1274-1279.1982. 6804441 PMC216350

[25] Porter RD, Welliver RA, Witkowski TA. 1982. Specialized transduction with lambda plac5: dependence on recB. J Bacteriol 150:1485–1488. doi:10.1128/jb.150.3.1485-1488.1982. 6210690 PMC216379

[26] Kleiner M, Bushnell B, Sanderson KE, Hooper LV, Duerkop BA. 2020. Transductomics: sequencing-based detection and analysis of transduced DNA in pure cultures and microbial communities. Microbiome 8:158. doi:10.1186/s40168-020-00935-5. 33190645 PMC7667829

[27] Chiang YN, Penadés JR, Chen J. 2019. Genetic transduction by phages and chromosomal islands: the new and noncanonical. PLoS Pathog 15:e1007878. doi:10.1371/journal.ppat.1007878. 31393945 PMC6687093

[28] Fey PD, Endres JL, Yajjala VK, Widhelm TJ, Boissy RJ, Bose JL, Bayles KW. 2013. A genetic resource for rapid and comprehensive phenotype screening of nonessential Staphylococcus aureus genes. mBio 4:e00537-12. doi:10.1128/mBio.00537-12. 23404398 PMC3573662

[29] Lee CY, Iandolo JJ. 1988. Structural analysis of staphylococcal bacteriophage phi 11 attachment sites. J Bacteriol 170:2409–2411. doi:10.1128/jb.170.5.2409-2411.1988. 2966144 PMC211141

[30] Goerke C, Pantucek R, Holtfreter S, Schulte B, Zink M, Grumann D, Bröker BM, Doskar J, Wolz C. 2009. Diversity of prophages in dominant Staphylococcus aureus clonal lineages. J Bacteriol 191:3462–3468. doi:10.1128/JB.01804-08. 19329640 PMC2681900

[31] Campoy S, Hervàs A, Busquets N, Erill I, Teixidó L, Barbé J. 2006. Induction of the SOS response by bacteriophage lytic development in Salmonella enterica. Virology 351:360–367. doi:10.1016/j.virol.2006.04.001. 16713610

[32] Ebel-Tsipis J, Fox MS, Botstein D. 1972. Generalized transduction by bacteriophage P22 in Salmonella typhimurium. II. Mechanism of integration of transducing DNA. J Mol Biol 71:449–469. doi:10.1016/0022-2836(72)90362-2. 4564487

[33] Haaber J, Leisner JJ, Cohn MT, Catalan-Moreno A, Nielsen JB, Westh H, Penadés JR, Ingmer H. 2016. Bacterial viruses enable their host to acquire antibiotic resistance genes from neighbouring cells. Nat Commun 7:13333. doi:10.1038/ncomms13333. 27819286 PMC5103068

[34] Bae T, Class EM, Schneewind O, Missiakas D. 2007. Generating a collection of insertion mutations in the Staphylococcus aureus genome using bursa aurealis. Methods Mol Biol 416:103–116. doi:10.1007/978-1-59745-321-9_7. 18392963

[35] Page AJ, De Silva N, Hunt M, Quail MA, Parkhill J, Harris SR, Otto TD, Keane JA. 2016. Robust high-throughput prokaryote de novo assembly and improvement pipeline for Illumina data. Microb Genom 2:e000083. doi:10.1099/mgen.0.000083. 28348874 PMC5320598

[36] Ponstingl H. 2016. SMALT – Wellcome Sanger Institute, http://sourceforge.net/projects/smalt/.

[37] Carver T, Harris SR, Berriman M, Parkhill J, Mcquillan JA. 2012. Artemis: an integrated platform for visualization and analysis of high-throughput sequence-based experimental data. Bioinformatics 28:464–469. doi:10.1093/bioinformatics/btr703. 22199388 PMC3278759

[38] Heringa S, Monroe J, Herrick J. 2007. A Simple, Rapid Method for Extracting Large Plasmid DNA from Bacteria. Nat Prec doi:10.1038/npre.2007.1249.1.

