# Supplementary material for: Screening for Highly Transduced Genes in Staphylococcus aureus Revealed Both Lateral and Specialized Transduction
Source: Microbiol Spectr. 2022 Feb 9;10(1):e02423-21. doi: 10.1128/spectrum.02423-21 (PMC8826898; doi:10.1128/spectrum.02423-21)
Supplement: SUPPLEMENTAL FILE 1 — Supplemental material. Download SPECTRUM02423-21_Supp_1_seq13.pdf, PDF file, 1.5 MB [file spectrum02423-21_supp_1_seq13.pdf]

<u>Supplementary figures and tables</u>

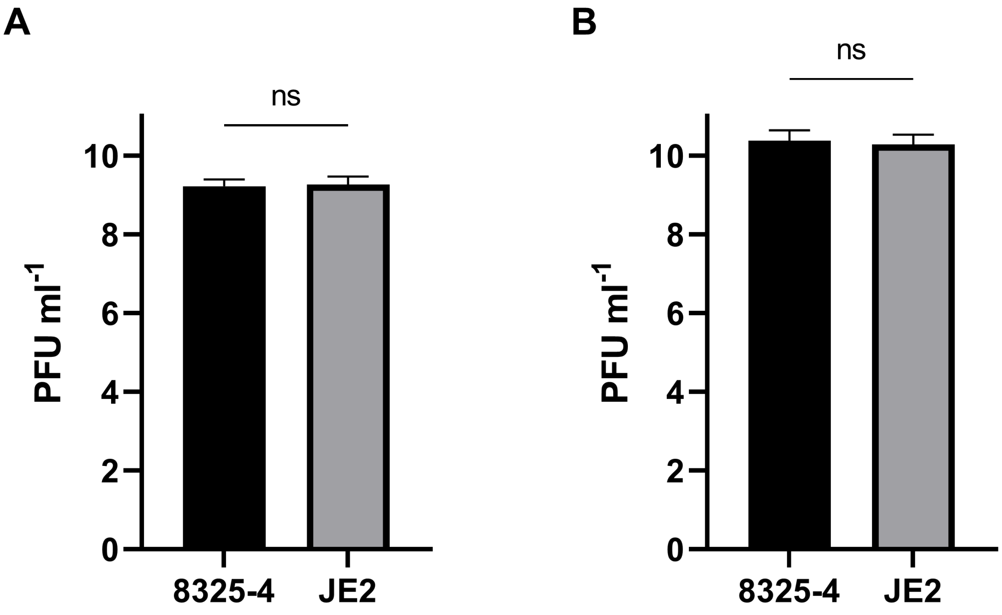

Supplementary Figure 1. Phage titre of ϕ11 following solid and liquid infection of 8325-4 and JE2. A. PFU ml^{-1} titre of an induced ϕ11 lysate using either 8325-4 or JE2 as the recipient. P value following unpaired t test, 0.7765^{ns}. **B.** PFU ml^{-1} titre of a ϕ11 lysate quantified on 8325-4, following liquid infection of either 8325-4 or JE2. P value following unpaired t test, 0.6677^{ns}. For both graphs, the values shown have been log transformed and show the mean and standard deviation (SD) for 3 biological replicates.

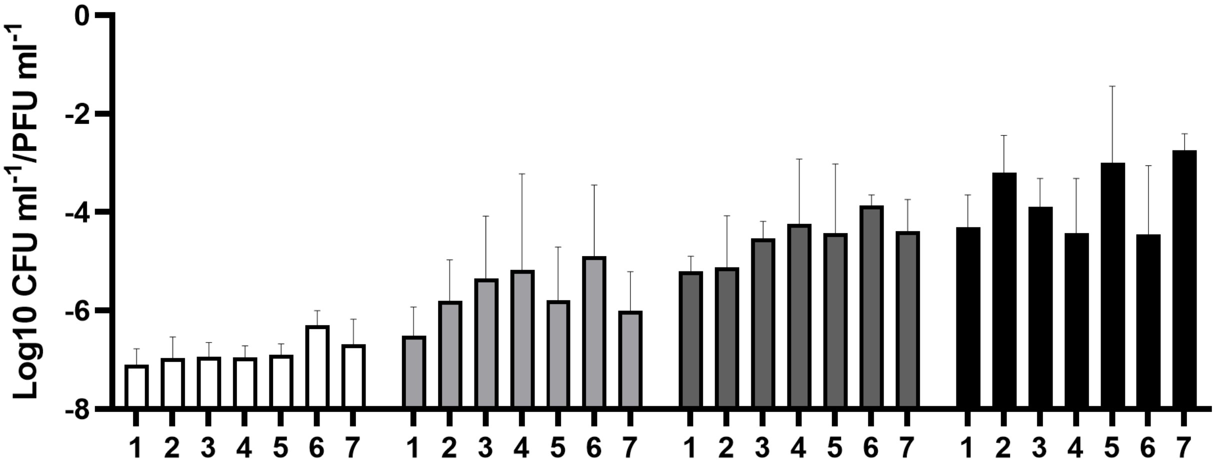

Supplementary Figure 2. The transduction frequencies of transduction-evolved, pooled transposon mutants. Transduction frequencies (log transformed CFU ml^{-1}/PFU ml^{-1}) for the initial infection transduction (white), the rounds 1 (light grey), 2 (dark grey), and 3 (black) induction-transduction. CFU ml^{-1} was calculated from the number of transductant colonies on erythromycin selective plates and PFU ml^{-1} from the number of plaques formed on a susceptible recipient (8325-4). Each bar represents the mean of three biological replicates of a pool of 96 transposon mutants labelled by plate number (1-7) with SD. A two-way ANOVA was performed with Tukey's correction for multiple comparisons, comparing the means of each round of transduction. There was no significant variation for the plates (p-value 0.2801), whilst the rounds showed significant variation overall (p-value, $<0.0001^{****}$). The p-values between successive transduction rounds were as follows, infection – round 1: 0.0004^{***}, round 1 – 2: 0.0010^{**}, round 2 – 3: 0.0230^{*}.

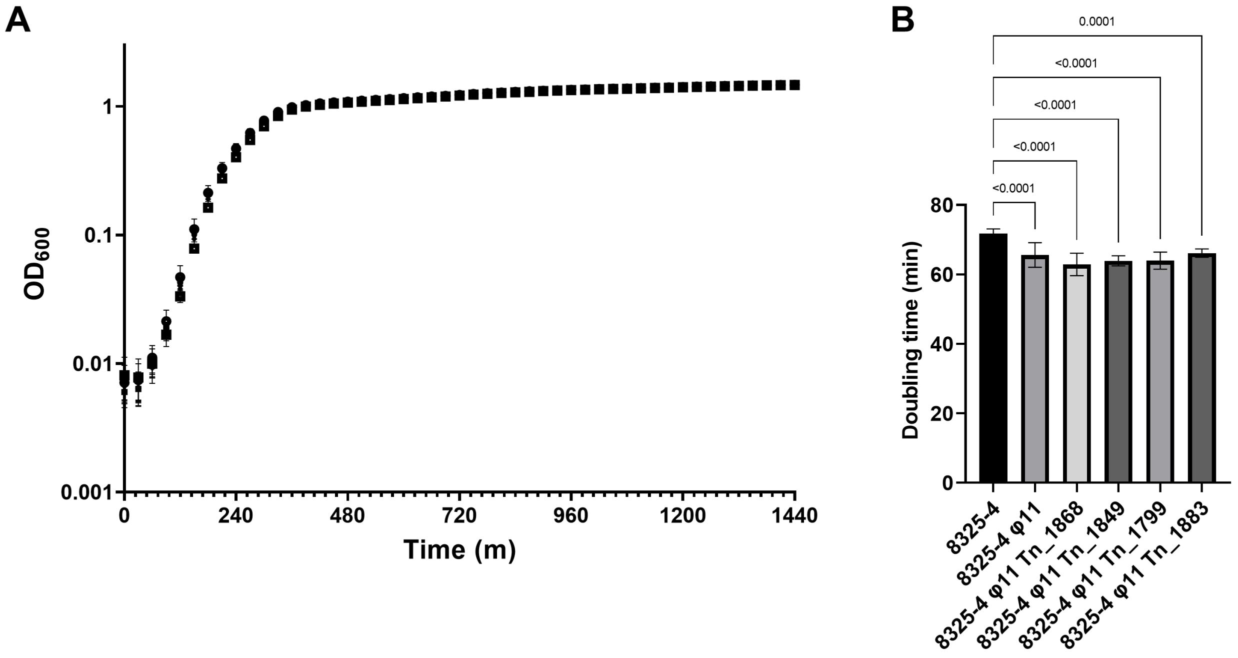

Supplementary figure 3. Growth curves of various 8325-4 ϕ11 transposon mutants and controls. A. Raw OD_{600} values for strains 8325-4 (black circle), 8325-4 ϕ11 (square), 8325-4 ϕ11 Tn_1868 (inverted triangle), 8325-4 ϕ11 Tn_1849 (diamond), 8325-4 ϕ11 Tn_1799 (white circle), and 8325-4 ϕ11 Tn_1883 (white square) when grown in TSB at 37 °C for 24 hours. Graph shows the mean and SD data for three biological replicates, each with three triplicate replicates included. **B.** The doubling time for the different strains when analysed using growthcurver. Doubling times were compared using a one-way ANOVA with Tukey's correction for multiple comparisons, significant differences are indicated on the graph and all p-values can be seen in Supplementary table 3.

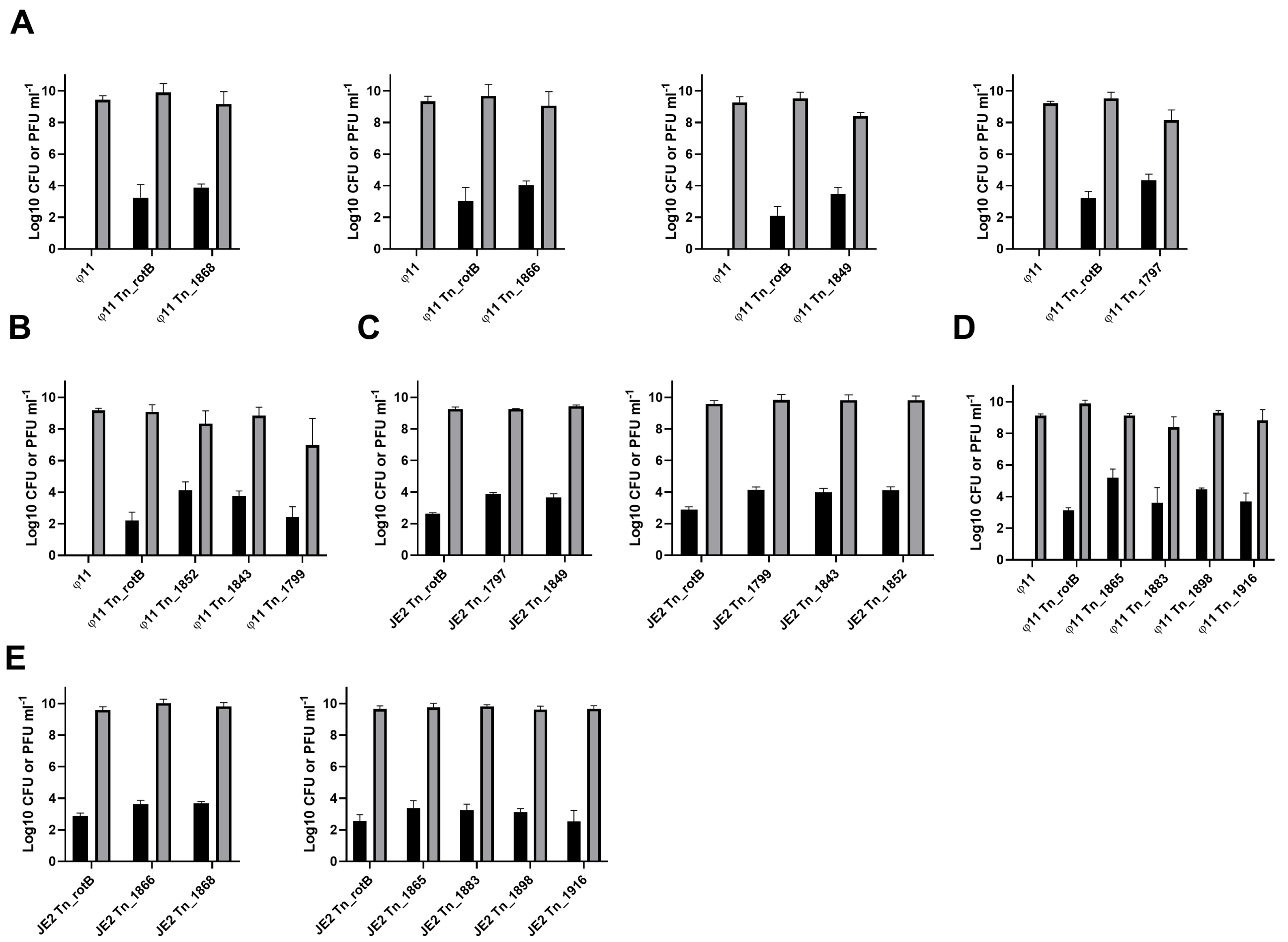

Supplementary figure 4. The numbers of phage and transductants from lysates of transposon mutants located up- and downstream of the ϕ11 integration site. A shows the CFU ml^{-1} (black) and PFU ml^{-1} (grey) for control strains 8325-4 ϕ11 and 8325-4 ϕ11 Tn_rotB with the 4 different Tn mutants up- and downstream of the ϕ11 integration site (Tn inserted into SAUSA300_1868, SAUSA300_1866, SAUSA300_1849, and SAUSA300_1797 respectively), related to Fig 3. **B** shows the CFU ml^{-1} and PFU ml^{-1} for control strains 8325-4 ϕ11 and 8325-4 ϕ11 Tn_rotB with the 3 downstream Tn mutations referred to in Fig 4 (Tn inserted into SAUSA300_1852, SAUSA300_1843, and SAUSA300_1799 respectively). The

values shown for A and B have been log transformed and show the mean and standard deviation (SD) for 4 biological replicates. **C** CFU ml^{-1} and PFU ml^{-1} for the control JE2 Tn_rotB and selected mutants, related to Fig 5. Left; the screen-identified Tn mutants downstream of the ϕ11 integration site following infection with ϕ11 (Tn inserted into SAUSA300_1797 and SAUSA300_1849 respectively), right; the other Tn mutants downstream of the ϕ11 integration site following infection with ϕ11 (Tn inserted into SAUSA300_1799, SAUSA300_1843 and SAUSA300_1852 respectively). Values shown have been log transformed and show the means and SD from the 3 replicate values. **D** shows the CFU ml^{-1} and PFU ml^{-1} for the controls 8325-4 ϕ11 and 8325-4 ϕ11 Tn_rotB with 4 different Tn mutations inserted separately in genes upstream of the ϕ11 integration site (Tn inserted into SAUSA300_1865, SAUSA300_1883, SAUSA300_1898 and SAUSA300_1916 respectively, progressively getting further away from the ϕ11 insertion site from left to right), related to Fig 6. All values shown have been log transformed and show the means and SD for 3 biological replicates. **E** The CFU ml^{-1} and PFU ml^{-1} of upstream JE2 transposon mutants following infection with ϕ11, related to Fig 7. Left, shows the CFU ml^{-1} and PFU ml^{-1} for the control JE2 Tn_rotB and the different screen-identified Tn mutants upstream of the ϕ11 integration site following infection with ϕ11 (Tn inserted into SAUSA300_1866 and SAUSA300_1868 respectively). Right, CFU ml^{-1} and PFU ml^{-1} for the control JE2 Tn_rotB and the different neighbouring Tn mutants upstream of the ϕ11 integration site following infection with ϕ11 (Tn inserted into SAUSA300_1865, SAUSA300_1883, SAUSA300_1898 and SAUSA300_1916 respectively).

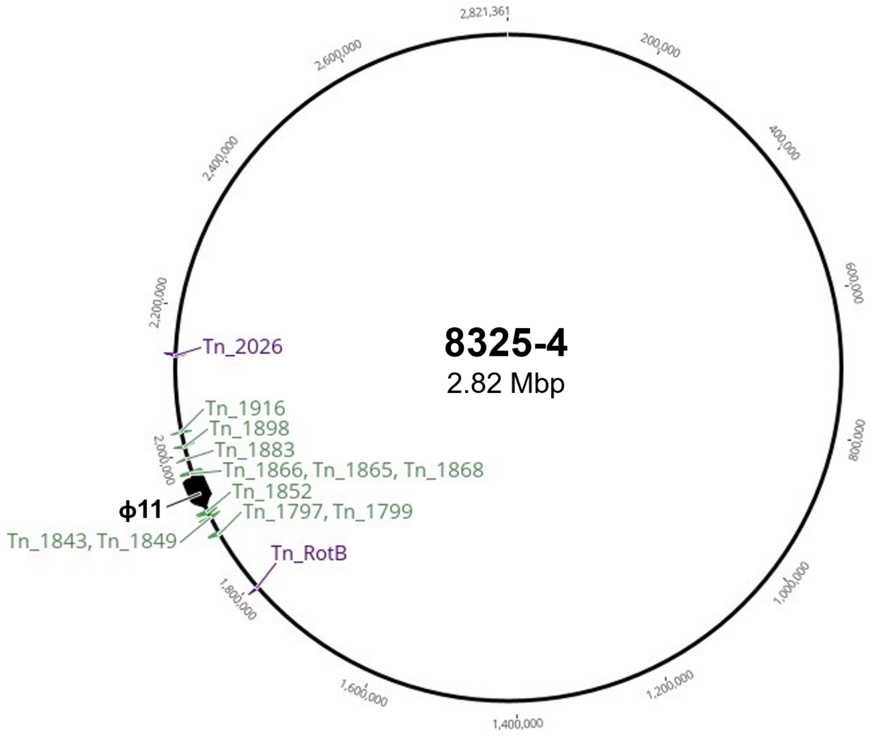

Supplementary Figure 5. Schematic drawing of the transposon mutants used in figures 3 – 7. Figure shows a schematic drawing of the 8325 genome indicating the location and direction of the ϕ11 prophage and the locations of all the transposon mutants included in figures 3 – 7, together with the Tn_RotB control and Tn_2026 from supplementary figure 6.

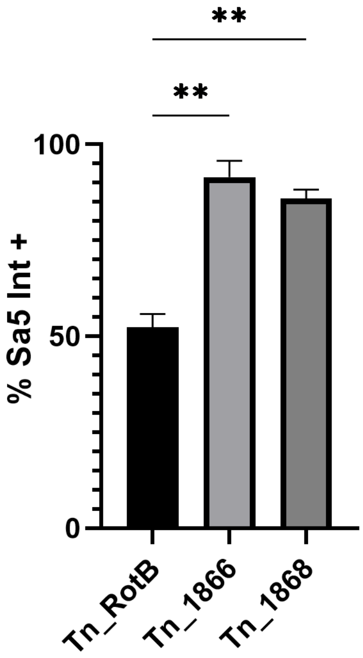

Supplementary Figure 6. PCR analysis of Sa5 integrase in 8325-4 Tn_1866 and Tn_1868 transductants. Shows the percentage of Sa5 integrase positive colonies when screening 8325-4 transductants of 8325-4 recipients of either ϕ11 Tn_RotB, Tn_1866 or Tn_1868 lysates. Data shown is the means of 2 biological replicates screening 96 colonies per replicate, with SD. P values comparing the Tn_1866 and tn_1868 to the Tn_RotB control with a one-way ANOVA with Dunnett corrections for multiple comparisons were 0.0024** and 0.0038** respectively.

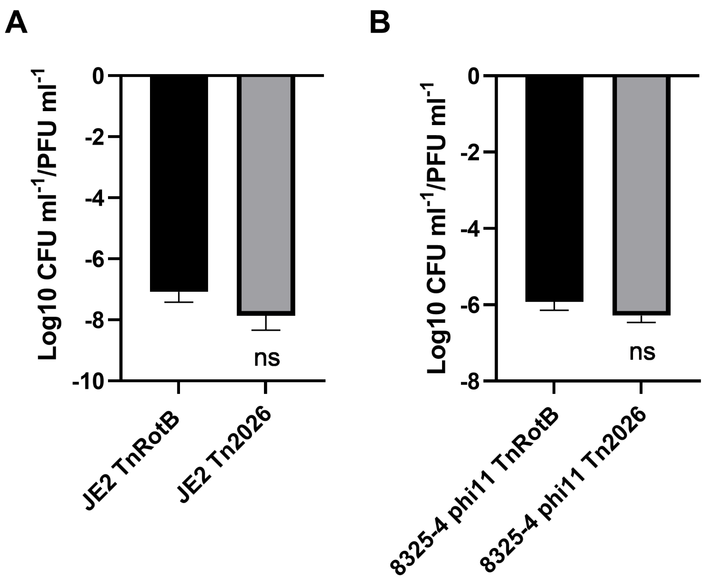

Supplementary Figure 7. The transduction frequency of an upstream transposon mutation outside the region of elevated reads mapping. A, shows the CFU ml^{-1} /PFU ml^{-1} transduction frequency for the control JE2 Tn_rotB and the JE2 Tn_2026 (located ~169 kb upstream of the ϕ11 integration site) following infection with ϕ11. The p value of an unpaired t test was 0.0816^{ns}. **B**, CFU ml^{-1} /PFU ml^{-1} transduction frequency for the same mutations in the 8325-4 φ11 background following phage induction. The p value of an unpaired t test was 0.0924^{ns}. All values shown have been log transformed and show the means and standard deviation from the 3 replicate values.

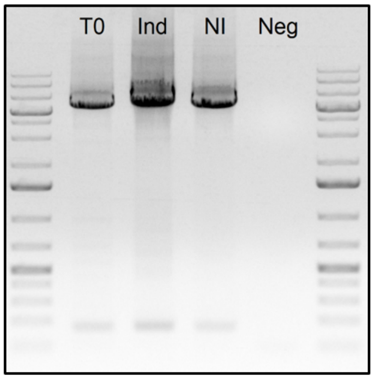

Supplementary Figure 8. Amplified circularisation based on homologous regions flanking the ϕ11 prophage. 0.7% agarose gel showing the PCR product of amplification using DreamsTaq polymerase. DNA from a large plasmid extraction of 8325-4 ϕ11, with or without phage induction. Three biological replicates were performed including induction/infection, DNA extraction and PCR, and the above gel is representative. T0, sample from time point 0; Ind/I, sample from 60 min following phage induction; NI, sample from 60 min without phage induction/infection; Neg, negative control. Primers used are listed in Supplementary Table 2; Homology check 1F and Homology check 2R.

Supplementary Table 1. Bacterial strains used in study.

Description	Reference
Phage-cured 8325 *S. aureus* strain, 8325-4	(1)
Phage-cured 8325 *S. aureus* strain with prophage ϕ11, 8325-4 ϕ11	(2)
8325-4 ϕ11 with NARSA transposon inserted in gene SAUSA300_1708/RotB.	This work
8325-4 ϕ11 with NARSA transposon inserted in gene SAUSA300_0810.	This work
8325-4 ϕ11 with NARSA transposon inserted in gene SAUSA300_1430.	This work
8325-4 ϕ11 with NARSA transposon inserted in gene SAUSA300_1797.	This work
8325-4 ϕ11 with NARSA transposon inserted in gene SAUSA300_1799.	This work
8325-4 ϕ11 with NARSA transposon inserted in gene SAUSA300_1843.	This work
8325-4 ϕ11 with NARSA transposon inserted in gene SAUSA300_1849.	This work
8325-4 ϕ11 with NARSA transposon inserted in gene SAUSA300_1852.	This work
8325-4 ϕ11 with NARSA transposon inserted in gene SAUSA300_1865.	This work
8325-4 ϕ11 with NARSA transposon inserted in gene SAUSA300_1866.	This work
8325-4 ϕ11 with NARSA transposon inserted in gene SAUSA300_1868.	This work
8325-4 ϕ11 with NARSA transposon inserted in gene SAUSA300_1883.	This work
8325-4 ϕ11 with NARSA transposon inserted in gene SAUSA300_1898.	This work
8325-4 ϕ11 with NARSA transposon inserted in gene SAUSA300_1916.	This work
8325-4 ϕ11 with NARSA transposon inserted in gene SAUSA300_1958.	This work
8325-4 ϕ11 with NARSA transposon inserted in gene SAUSA300_1971.	This work
8325-4 ϕ11 with NARSA transposon inserted in gene SAUSA300_2026.	This work
JE2	(3)
JE2 with NARSA transposon inserted in gene SAUSA300_1708/RotB (NTML 96-plate 2.1, well A2).	(4)
JE2 with NARSA transposon inserted in gene SAUSA300_0810 (NTML 96-plate 3.4, well B5).	(4)

JE2 with NARSA transposon inserted in gene SAUSA300_1430 (NTML 96-plate 4.3, well F4).	(4)
JE2 with NARSA transposon inserted in gene SAUSA300_1797 (NTML 96-plate 4.4, well A5).	(4)
JE2 with NARSA transposon inserted in gene SAUSA300_1799 (NTML 96-plate 3.3, well H9).	(4)
JE2 with NARSA transposon inserted in gene SAUSA300_1843 (NTML 96-plate 4.4, well B8).	(4)
JE2 with NARSA transposon inserted in gene SAUSA300_1849 (NTML 96-plate 3.3, well G8).	(4)
JE2 with NARSA transposon inserted in gene SAUSA300_1852 (NTML 96-plate 3.3, well B5).	(4)
JE2 with NARSA transposon inserted in gene SAUSA300_1865 (NTML 96-plate 2.2, well G2).	(4)
JE2 with NARSA transposon inserted in gene SAUSA300_1866 (NTML 96-plate 3.1, well E7).	(4)
JE2 with NARSA transposon inserted in gene SAUSA300_1868 (NTML 96-plate 3.2, well D7).	(4)
JE2 with NARSA transposon inserted in gene SAUSA300_1883 (NTML 96-plate 3.2, well C1).	(4)
JE2 with NARSA transposon inserted in gene SAUSA300_1898 (NTML 96-plate 1.3, well F9).	(4)
JE2 with NARSA transposon inserted in gene SAUSA300_1916 (NTML 96-plate 3.1, well E12).	(4)
JE2 with NARSA transposon inserted in gene SAUSA300_1958 (NTML 96-plate 3.1, well E8).	(4)
JE2 with NARSA transposon inserted in gene SAUSA300_1971 (NTML 96-plate 4.1, well H12).	(4)
JE2 with NARSA transposon inserted in gene SAUSA300_2026 (NTML 96-plate 5.4, well A9).	(4)

Description	Reference
96 strains from NTML 96-plate named 3.1	(4)
96 strains from NTML 96-plate named 3.2	(4)
96 strains from NTML 96-plate named 3.3	(4)
96 strains from NTML 96-plate named 3.4	(4)
96 strains from NTML 96-plate named 4.1	(4)
96 strains from NTML 96-plate named 4.2	(4)
96 strains from NTML 96-plate named 4.3	(4)
96 strains from NTML 96-plate named 4.4	(4)

Supplementary Table 2. Primers used in this study.

Oligonucleotides	Sequences (5’-3’)
Buster	GCTTTTTCTAAATGTTTTTTAAGTAAATCAAGTACC
Martn-ermR	AAACTGATTTTTAGTAAACAGTTGACGATATTC
Sa1-F	AAGCTAAGTTCGGGCACA
Sa1-R	GTAATGTTTGGGAGCCAT
Sa2-F	TCAAGTAACCCGTCAACTC
Sa2-R	ATGTCTAAATGTGTGCGTG
Sa3-F	GAAAAACAAACGGTGCTAT
Sa3-R	TTATTGACTCTACAGGCTGA
Sa4-F	ATTGATATTAACGGAACTC
Sa4-R	TAAACTTATATGCGTGTGT
Sa5-F	AAAGATGCCAAACTAGCTG
Sa5-R	CTTGTGGTTTTGTTCTGG
Sa6-F	GCCATCAATTCAAGGATAG
Sa6-R	TCTGCAGCTGAGGACAAT
Sa7-F	GTCCGGTAGCTAGAGGTC
Sa7-R	GGCGTATGCTTGACTGTGT
Phage direction-3f	CGTATCATTTTAAATCTGTATTCCCG
Ribo check 1F	CGGCTCATCGCATCCACTTTTTGCC
Ribo check 2R	GTAACACATCATCGAATGGAAATTTATGGAGTTTG

Supplementary Table 3. P-values for comparisons between strain doubling times in Supplementary figure 2.

Number of families	1
Number of comparisons per family	15
Alpha	0.05

Tukey's multiple comparisons test	Mean Diff.	95.00% CI of diff.	Below threshold?	Summary	Adjusted P Value
8325-4 vs. 8325-4 ϕ11	6.119	2.764 to 9.473	Yes	****	<0.0001
8325-4 vs. 8325-4 ϕ11 Tn_1868	8.861	5.507 to 12.22	Yes	****	<0.0001
8325-4 vs. 8325-4 ϕ11 Tn_1849	7.803	4.449 to 11.16	Yes	****	<0.0001
8325-4 vs. 8325-4 ϕ11 Tn_1799	7.735	4.381 to 11.09	Yes	****	<0.0001
8325-4 vs. 8325-4 ϕ11 Tn_1883	5.607	2.253 to 8.961	Yes	***	0.0001
8325-4 ϕ11 vs. 8325-4 ϕ11 Tn_1868	2.743	-0.6117 to 6.097	No	ns	0.1677
8325-4 ϕ11 vs. 8325-4 ϕ11 Tn_1849	1.685	-1.670 to 5.039	No	ns	0.6717
8325-4 ϕ11 vs. 8325-4 ϕ11 Tn_1799	1.617	-1.737 to 4.971	No	ns	0.7085
8325-4 ϕ11 vs. 8325-4 ϕ11 Tn_1883	-0.5115	-3.866 to 2.843	No	ns	0.9975
8325-4 ϕ11 Tn_1868 vs. 8325-4 ϕ11 Tn_1849	-1.058	-4.412 to 2.296	No	ns	0.9351

8325-4 ϕ11 Tn_1868 vs. 8325-4 ϕ11 Tn_1799	-1.126	-4.480 to 2.229	No	ns	0.917
8325-4 ϕ11 Tn_1868 vs. 8325-4 ϕ11 Tn_1883	-3.254	-6.608 to 0.1002	No	ns	0.062
8325-4 ϕ11 Tn_1849 vs. 8325-4 ϕ11 Tn_1799	-0.06772	-3.422 to 3.287	No	ns	>0.9999
8325-4 ϕ11 Tn_1849 vs. 8325-4 ϕ11 Tn_1883	-2.196	-5.550 to 1.158	No	ns	0.3895
8325-4 ϕ11 Tn_1799 vs. 8325-4 ϕ11 Tn_1883	-2.128	-5.483 to 1.226	No	ns	0.4246

1. O'Neill AJ. 2010. Staphylococcus aureus SH1000 and 8325-4: comparative genome sequences of key laboratory strains in staphylococcal research. Letters in Applied Microbiology 51:358–361.

2. Iandolo JJ, Worrell V, Groicher KH, Qian Y, Tian R, Kenton S, Dorman A, Ji H, Lin S, Loh P, Qi S, Zhu H, Roe BA. 2002. Comparative analysis of the genomes of the temperate bacteriophages φ11, φ12 and φ13 of Staphylococcus aureus 8325. Gene 289:109–118.

3. Bae T, Class EM, Schneewind O, Missiakas D. 2007. Generating a collection of insertion mutations in the Staphylococcus aureus genome using bursa aurealis. Methods in Molecular Biology 416:103–116.

4. Fey PD, Endres JL, Yajjala VK, Widhelm TJ, Boissy RJ, Bose JL, Bayles KW. 2013. A genetic resource for rapid and comprehensive phenotype screening of nonessential Staphylococcus aureus genes. mBio 4.
